# The effect of psychological interventions on anxiety and depression in cancer patients: results of two meta-analyses

**DOI:** 10.1038/sj.bjc.6690596

**Published:** 1999-08

**Authors:** T Sheard, P Maguire

**Affiliations:** CAA Bristol Oncology Centre, Horfield Road, Bristol, BS2 8ED, UK; CRC Psychological Medicine Group, Christie CRC Research Centre, Wilmslow Road, Manchester, M20 4BX, UK

**Keywords:** cancer, psychological interventions, counselling, psychotherapy, anxiety, depression, meta-analysis

## Abstract

The findings of two meta-analyses of trials of psychological interventions in patients with cancer are presented: the first using anxiety and the second depression, as a main outcome measure. The majority of the trials were preventative, selecting subjects on the basis of a cancer diagnosis rather than on psychological criteria. For *anxiety*, 25 trials were identified and six were excluded because of missing data. The remaining 19 trials (including five unpublished) had a combined effect size of 0.42 standard deviations in favour of treatment against no-treatment controls (95% confidence interval (CI) 0.08–0.74, total sample size 1023). A most robust estimate is 0.36 which is based on a subset of trials which were randomized, scored well on a rating of study quality, had a sample size > 40 and in which the effect of trials with very large effects were cancelled out. For *depression*, 30 trials were identified, but ten were excluded because of missing data. The remaining 20 trials (including six unpublished) had a combined effect size of 0.36 standard deviations in favour of treatment against no-treatment controls (95% CI 0.06–0.66, sample size 1101). This estimate was robust for publication bias, but not study quality, and was inflated by three trials with very large effects. A more robust estimate of mean effect is the clinically weak to negligible value of 0.19. Group therapy is at least as effective as individual. Only four trials targeted interventions at those identified as at risk of, or suffering significant psychological distress, these were associated with clinically powerful effects (trend) relative to unscreened subjects. The findings suggest that preventative psychological interventions in cancer patients may have a moderate clinical effect upon anxiety but not depression. There are indications that interventions targeted at those at risk of or suffering significant psychological distress have strong clinical effects. Evidence on the effectiveness of such targeted interventions and of the feasibility and effects of group therapy in a European context is required. © 1999 Cancer Research Campaign


*British Journal of Cancer* (1999) **80**(11), 1770–1780
© 1999 Cancer Research Campaign
Article no. bjoc.1999.0596

### The effect of psychological interventions on anxiety and depression in cancer patients: results of two meta-analyses

**T Sheard[1] and P Maguire[2]**

[1]CAA Bristol Oncology Centre, Horfield Road, Bristol BS2 8ED, UK; [2]CRC Psychological Medicine Group, Christie CRC Research Centre, Stanley House, Wilmslow Road, Manchester M20 4BX, UK

**Summary** The findings of two meta-analyses of trials of psychological interventions in patients with cancer are presented: the first using anxiety and the second depression, as a main outcome measure. The majority of the trials were preventative, selecting subjects on the basis of a cancer diagnosis rather than on psychological criteria. For *anxiety*, 25 trials were identified and six were excluded because of missing data. The remaining 19 trials (including five unpublished) had a combined effect size of 0.42 standard deviations in favour of treatment against no-treatment controls (95% confidence interval (CI) 0.08–0.74, total sample size 1023). A most robust estimate is 0.36 which is based on a subset of trials which were randomized, scored well on a rating of study quality, had a sample size > 40 and in which the effect of trials with very large effects were cancelled out. For *depression*, 30 trials were identified, but ten were excluded because of missing data. The remaining 20 trials (including six unpublished) had a combined effect size of 0.36 standard deviations in favour of treatment against no-treatment controls (95% CI 0.06–0.66, sample size 1101). This estimate was robust for publication bias, but not study quality, and was inflated by three trials with very large effects. A more robust estimate of mean effect is the clinically weak to negligible value of 0.19. Group therapy is at least as effective as individual. Only four trials targeted interventions at those identified as at risk of, or suffering significant psychological distress, these were associated with clinically powerful effects (trend) relative to unscreened subjects. The findings suggest that preventative psychological interventions in cancer patients may have a moderate clinical effect upon anxiety but not depression. There are indications that interventions targeted at those at risk of or suffering significant psychological distress have strong clinical effects. Evidence on the effectiveness of such targeted interventions and of the feasibility and effects of group therapy in a European context is required.

**Keywords**: cancer; psychological interventions; counselling; psychotherapy; anxiety; depression; meta-analysis

Between 15 and 40% of cancer patients develop clinical anxiety and/or depression (Derogatis et al, 1983; Massie and Holland, 1990; Parle et al, 1996). Even for those ostensibly cured the prevalence remains appreciably higher than that of the general population a year or more after diagnosis (Devlen et al, 1987). This evidence, combined with significant pressure from service users, has led to increasing provision of psychological interventions in British oncology services. This has been piecemeal (Fallowfield, 1988) and guided more by local factors than evidence of efficacy. There is little evidence based consensus about optimal methods of intervention, appropriate standards for clinical practice and training, or whether service provision is best directed towards prevention or the treatment of disorder (Fallowfield and Roberts, 1992; Hopwood and Maguire, 1992; Brennan and Sheard, 1994).

The impact of psychological interventions in oncology on psychosocial, disease, symptom and treatment side-effect outcomes has been evaluated in a relatively large number of trials which vary considerably in their research questions, methodology, settings and results. Meyer and Mark (1995) aggregated all psychological outcomes for 45 such trials and reported a small mean effect size of 0.24 standard deviations for a single aggregate measure of psychological outcome. Their broad entry criteria ensured the inclusion of a large number of trials but the resulting extreme heterogeneity in terms of diversity of research questions and outcome measures render the meaning of this aggregate finding difficult to assess. We conducted, therefore, two meta-analyses of trials of interventions which sought to treat or prevent anxiety and/or depression. The relevance of the findings to clinical practice and service provision is assessed.

*Received 30 January 1998*
*Revised 29 January 1999*
*Accepted 20 February 1999*

*Correspondence to:* T Sheard

### METHODS

### Identical methods were used for the two meta-analyses

*Search strategy*

Medline, PsycLit and BIDS social sciences computerized databases were searched using the using the keywords cancer, counselling, psychotherapy, psychological therapy, group support/therapy, relaxation, imagery and visualization. Citations in identified papers and reviews (Watson, 1983; Cunningham, 1988; Vachon, 1988; Harman, 1991; Andersen, 1992; Trijsburg et al, 1992), Aslib. index to theses (keywords cancer, counselling and psychotherapy), and Comprehensive Dissertation Abstracts: Psychology (keyword cancer) were manually searched.

### Inclusion and exclusion criteria

Trials were included if they (a) evaluated psychosocial or psychiatric interventions aimed specifically at alleviating psychological distress in oncology subjects, (but excluded if the main focus was the reduction of physical symptoms, prolongation of survival, impact on immune parameters or reduction of peri-surgical distress); (b) had a control condition; and (c) had been published in a journal or indexed as a dissertation before January 1993 when this project was initiated. Resource constraints limited inclusion of trials to those available in an English language form through British library services.

Single group designs (i.e. those without a control group) were excluded. Restricting eligibility to randomized control trials was considered but at present evidence from psychotherapy meta-analyses suggests that randomization does not affect outcome. Lipsey and Wilson (1993) in their meta-analysis of 136 meta-analyses of psychological interventions identified only three factors which significantly influenced size of effect: single group designs (i.e. those with no control group) and small sample size trials had larger effects (publication bias), while placebo-controlled trials had smaller effects. The possible influence of different aspects of trial quality on our meta-analyses are explored in a sensitivity analysis. Excluding non-randomized trials would also have the disadvantage of reducing the representativeness of the sample and the statistical power.

### Coding

Data from published and unpublished reports were entered on a standardized coding form which included criteria for assessing and coding ambiguous information. Study features were coded under specific domains: independent variable (e.g. type of therapy, 'dose' of therapy), subject (e.g. prognosis) and setting variables, experimental method, dependent variables (i.e. anxiety and depression), and quality of reporting. A system for scoring aspects of study methodology was devised based on Cook and Campbell's (1979) four categories of threats to validity (available on request). Studies which used more reliable methods were identified using three factors: (i) use of randomization, (ii) falling into the top 75% on overall quality score, and (iii) sample size greater than 40.

Results were coded for 'post-tests' (outcomes measured immediately after completing the intervention) when performed, or for observations taken between 3 and 6 months after commencement of flexible length interventions.

### Outcome measures

For those trials using more than one anxiety or depression measure the result from only one instrument was selected to represent each domain. Commonly used measures were selected preferentially to increase comparability. Failing this, the data from the instrument with superior psychometric properties were included, or if this was similar, selection was random.

### Statistical methods

Effect sizes for continuous psychological data are normally represented in terms of standard deviation shift; thus a positive value represents a better outcome for the intervention group. Where possible the effect size 'g' was estimated as a standardized mean difference: this is the mean value for the intervention group *minus* mean value for control group *divided by* their pooled standard deviation. For studies not providing this data 'g' was estimated from precise statistical test values (Hedges and Olkin, 1985). Hedges and Olkin (1985) have shown that 'g' has a small sample bias, this is corrected into the unbiased estimator 'd' which is very similar to 'g' for large studies but smaller in small studies. Confidence intervals for the effect sizes of individual studies were based on estimates of conditional variance as given by Hedges and Olkin (1995). Prof R Schwarzer's meta-analysis programme was used (Frei Universitat Berlin, Germany).

In view of the broad entry criteria, descriptive focus and an expectation of considerable heterogeneity the more conservative random effects analysis was used (Cook and Campbell, 1979; Raudenbush, 1994) both in estimation of main effects and in exploration of the moderating effect of variables which were hypothesized to affect outcome (such as the amount of therapy given, the therapists' level of training and experience, or cancer prognosis). Fixed and random effects models address the problem of heterogeneity in different ways and have complementary strengths and shortcomings (Cook and Campbell, 1979; Thompson, 1993). Estimates of the main effects obtained using these models are compared in a sensitivity analysis.

Tests for interaction were used to see whether there was evidence of different size of effect in two or more groups. This was achieved by subtracting the sum of Q's (homogeneity statistics) for individual groups from the Q for the individual groups combined. This yields a $\chi^2$ value with $n-1$ degrees of freedom where $n$ is the number of groups.

Publication bias (the tendency for only studies with statistically significant results to be accepted for publication) presents a considerable threat to the representativeness of meta-analysis samples. This was estimated using three methods. The effect sizes for published and unpublished studies were compared, a smaller mean effect size for unpublished studies would be an indicator of publication bias. Funnel plots of sample size against effect size indicate if small sample size trials are inadequately represented

**Table 1** Trials used in the meta-analyses

| |
|---|
| *Anxiety meta-analysis only* |
| Carpenter, 1984 |
| Cumbia, 1985 |
| Davis, 1986 |
| Golonka, 1976 |
| Johnson, 1982 |
| *Depression meta-analysis only* |
| Guerrant, 1984 |
| Hayes, 1981 |
| Linn et al, 1982 |
| Price, 1982 |
| West, 1980 |
| Youssef, 1984 |
| *Anxiety and depression meta-analyses* |
| Bindemann et al, 1991 |
| Bloom et al, 1978 |
| Cain et al, 1986 |
| Christensen, 1983 |
| Decker et al, 1992 |
| Fawzy et al, 1990 |
| Frankel, 1985 |
| Greer et al, 1992 |
| Houts et al, 1986 |
| Hurst, 1986 |
| Maguire et al, 1980 |
| Spiegel et al, 1981 |
| Telch and Telch, 1986 |
| Worden and Weisman, 1984 |

© 1999 Cancer Research Campaign

**Table 2** Exclusions

| Author | Origin | Year | Intervention type | Intervention mode | Sample size (one experimental group and controls) | Anxiety: Estimated effect of inclusion on mean effect size | Depression: Estimated effect of inclusion on mean effect size |
|---|---|---|---|---|---|---|---|
| Farash (Thesis) | USA | 1977 | Brief therapy | Individual | 40 | Not measured | Deflate |
| Gordon | USA | 1980 | Education and counselling | Individual | 308 | Deflate | Deflate |
| Capone | USA | 1980 | Crisis intervention | Individual | 97 | * | * |
| Fairbanks (Thesis) | USA | 1981 | Simonton and Rational emotive therapy | Group | 24 | Not measured | Not evaluable |
| Heinrich | USA | 1985 | Psychoeducational course | Group | 70 | * | * |
| Watson (M) | UK | 1988 | Information /expression of emotion | Individual | 28 | Very little effect | Inflate |
| Bridge | UK | 1988 | Relaxation and imagery | Individual | 92 | Deflate | Deflate |
| Burton | UK | 1988 | Rogerian: single session | Individual | 42 | No effect | Deflate |
| Clacey | UK | 1988 | Information and counselling | Individual | 25 | Probably little or no effect | Probably little or no effect |
| Halttunen | Finland | 1992 | Focus on appraisal and coping | Group | 65 | Not measured | Not evaluable |

A very approximate estimate of effect size was obtained by (a) calculating effect sizes from data from selectively presented outcomes and adjusting this down according to the extent of incompletely reported data for other outcomes, (b) noting when outcomes hardly differed between treatment and control groups, (c) assuming small effect sizes in trials which had statistically non-significant results despite a large sample. *Low scores for distress at baseline and follow-up in treatment and control groups and therefore treatment effect not demonstrable.

while Rosenthal's (1979) 'fail safe *n*' indicates the number of unpublished studies of effect size zero locked away in researchers' filing cabinets which would be required to reduce the mean effect size to a specific level.

For those studies comparing two different interventions with a common control group the intervention groups' effect sizes are not independent and therefore the data for the less structured intervention arms were eliminated (e.g. in a comparison of a coping skills intervention with simple support the support arm was eliminated (Telch and Telch, 1986)).

### RESULTS

Anxiety was used as an outcome measure in 19 studies, depression in 20 and 14 studies were common to the two meta-analyses (Table 1). Five trials (Worden and Weisman, 1984; Cumbia, 1985; Cain et al, 1986; Davis, 1986; Telch and Telch, 1986) compared two types of intervention with a common control. Only three studies (Maguire et al, 1980; Bindemann et al, 1991; Greer et al, 1992), all of individual therapy, were conducted outside of the US, many were conducted in university hospitals and the samples were generally skewed towards whites, the well-educated, women and a diagnosis of breast cancer. Inclusion criteria were largely medical rather than psychological. The majority of trials were preventative in orientation: only three anxiety (Worden and Weisman, 1984; Telch and Telch, 1986; Greer et al, 1992) and four depression trials (West, 1980; Worden and Weisman, 1984; Telch and Telch, 1986; Greer et al, 1992) restricted inclusion to those identified as being at risk of, or suffering, significant psychological distress. In other trials subjects were recruited sequentially on the basis of a cancer diagnosis, or on referral by an oncologist, or were self-selected. The analyses of the anxiety and depression data are presented separately. Table 1 lists the references included in the anxiety and/or depression meta-analyses.

### Anxiety meta-analysis

Nineteen trials were included (Table 1). A total of 26 trials were identified measuring anxiety and met initial entry criteria, but six were excluded as effect sizes could not be estimated (Table 2). Figure 1 shows the spread and confidence intervals for the 19 studies. The total sample size is 1023, the combined effect size is 0.42 and the dataset is strongly heterogeneous. Nine trials used the Profile of Mood States (POMS) (McNair et al, 1971) tension subscale as a measure of anxiety, five used the Spielberger State Anxiety Inventory (Spielberger et al, 1983), and the remaining five used other measures.

#### *Sensitivity Analysis (Table 2 and Figure 2)*

Table 2 summarizes trials excluded from both meta-analyses. It was possible in some cases to gain an indication of the magnitude

 *© 1999 Cancer Research Campaign*

| Author & Year | | Treatment mode | *n** | R |
|---|---|---|---|---|
| Golonka (T) | 1976 | Group | 38 | – |
| Bloom | 1978 | Individ | 39 | – |
| Maguire | 1980 | Individ | 152 | + |
| Spiegel | 1981 | Group | 30 | + |
| Johnson | 1982 | Group | 52 | + |
| Christen. | 1983 | Couple | 20 | + |
| Carpent.(T) | 1984 | Group | 22 | + |
| Worden | 1984 | Individ | 117 | – |
| Cumbia (T) | 1985 | Group | 12 | + |
| Frankel (T) | 1985 | Group | 24 | + |
| Cain | 1986 | Individ | 72 | + |
| Davis | 1986 | Individ | 12 | + |
| Houts | 1986 | Individ | 32 | – |
| Hurst (T) | 1986 | Group | 23 | + |
| Telch | 1986 | Group | 27 | + |
| Fawzy | 1990 | Group | 66 | + |
| Bindem. | 1991 | Relax | 71 | + |
| Decker | 1992 | Relax | 63 | + |
| Greer | 1992 | Individ | 151 | + |
| **Total** | | | **1023** | |

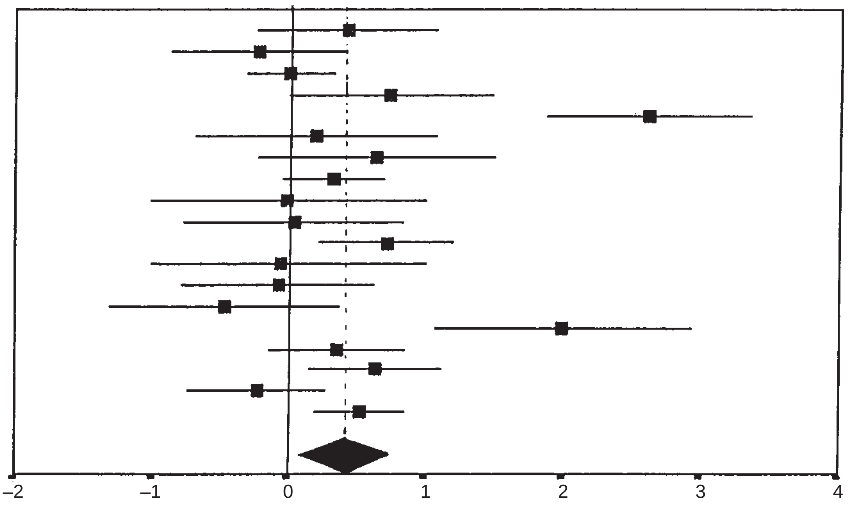

(T) = Unpublished thesis
R = Randomized assignment
* For trials with more than one intervention group (Cain, Davis and Telch) sample size is the number in the included experimental group plus the number in the control group

Effect size (95% confidence intervals)
(vertical solid line indicates zero and dotted line the mean effect size)

Mean effect size is 0.42 (95% CI 0.08–0.74)
Strongly Heterogenous: Q= 69.2175, P < 0.00000

**Figure 1** Effect sizes and 95% confidence intervals for individual studies: anxiety

| | *n** | ES** |
|---|---|---|
| Published | 14 | 0.51 |
| Unpublished | 5 | 0.16 |
| More reliable design | 8 | 0.63 |
| Less reliable design | 11 | 0.24 |
| Two extreme positive outliers removed | 17 | 0.27 |
| Fixed effects analysis | 19 | 0.36 |
| Random effects analysis | 19 | 0.42 |

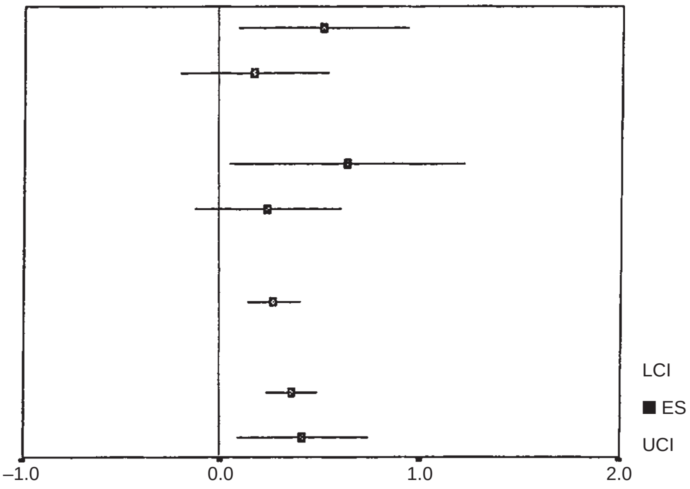

Effect size and 95% confidence intervals

* *n* = Number of trials
**ES = Effect size (All random effects analysis with exception of fixed effects statistic). Vertical line is zero exception of fixed effects statistic) Vertical line is zero

**Figure 2** Sensitivity analysis: anxiety of effect in the excluded studies. Inclusion of two (Gordon et al, 1980; Bridge et al, 1988) might have reduced the mean effect size for anxiety (see second last column Table 2).

Two extreme positive outliers stand out in Figure 1 (Johnson, 1982; Telch and Telch, 1986) and have a combined sample size of 79. Removal of these reduces the effect size by a third to 0.27 (Figure 2).

There are indications that the use of randomization and other features contributing to greater reliability in design are associated with larger effects (Figure 2). Fifteen studies used randomization to determine allocation to condition and these have a greater combined effect size than the four non-randomized studies (0.5 vs 0.19). Eight studies met our criteria for greater reliability and these have a greater mean effect size than the 11 studies of less reliable design (0.63 vs 0.24). This difference is not attributable to the two extreme positive outliers as one fell into each group. Removing the one positive outlier (effect size 2.6, sample size 52) (Johnson, 1982) from the group of 11 trials of more reliable design reduces the effect size by nearly 50% to 0.36 (95% CI 0.095–0.63).

| | $n^1$ | $ES^2$ | $P^3$ |
|---|---|---|---|
| Individual therapy | 8 | 0.27 | |
| Relaxation | 2 | 0.21 | 0.0076 df = 2 |
| Group therapy | 9 | 0.69 | |
| Group therapy excluding psycho-education | 6 | 0.27 | 0.0005 df = 1 |
| Group psycho-education | 3 | 1.59 | |
| All studies: Anxiety | 19 | 0.42 | 0.0000 |

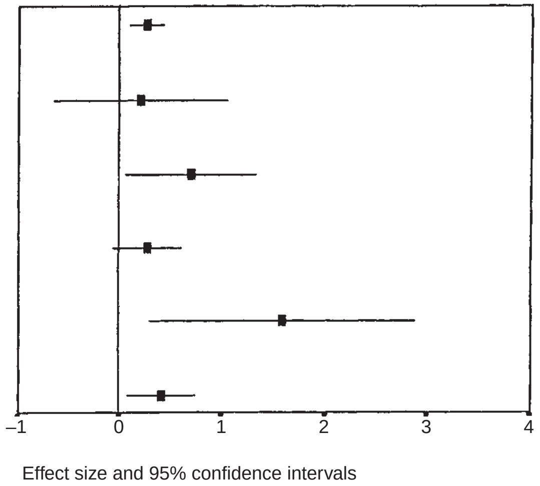

Vertical line represents mean effect size

[1]n = Number of trials
[2]ES = Effect size (random effects analysis)
[3]*P* = *P* value for heterogeneity test between variables (random effects analysis)

**Figure 3** Treatment type: anxiety

| | $n^1$ | $ES^2$ | $P^3$ |
|---|---|---|---|
| <4 hours therapy | 4 | 0.21 | |
| 4–7 hours therapy | 6 | 0.41 | 0.0017 |
| 8+ hours therapy | 4 | 1.01 | df=2 |
| Less experienced therapist | 4 | 0.10 | |
| More experienced therapist | 6 | 0.57 | 0.0540 |
| Advanced disease | 3 | 0.46 | |
| Good or mixed prognosis | 13 | 0.4 | 0.28 |
| Screened | 3 | 0.85 | |
| Not screened | 5 | 0.33 | 0.1715 |
| All studies anxiety | 19 | 0.42 | 0.0000 |

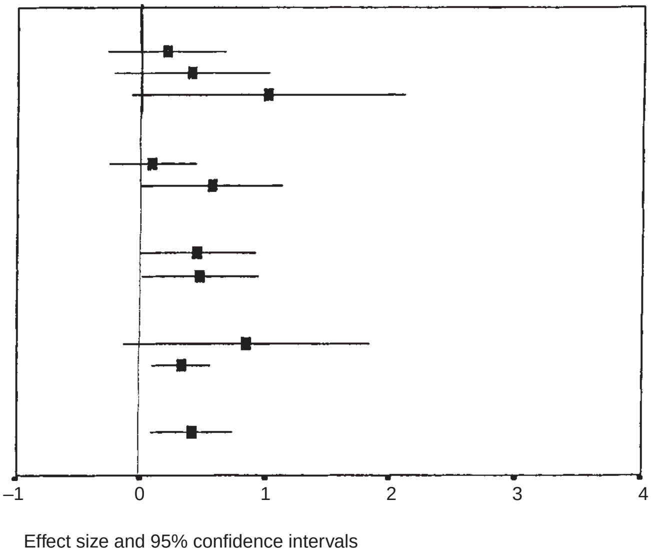

Vertical line represents mean effect size (random effects analysis)

[1]n = Number of trials
[2]ES = Effect size (random effects analysis)
[3]*P* = *P* value for heterogeneity test between variables

**Figure 4** Dose, therapist and subject variables: anxiety

The data from this sample suggest bias in the published sub-sample: published studies (*n* = 14) have a mean effect size of 0.51, compared to 0.16 for unpublished theses (*n* = 5). However, including the unpublished theses in a funnel plot results in a reasonably symmetrical distribution. Applying Rosenthal's 'fail safe *n*' indicates that 20 undetected studies of effect size zero are required to reduce the effect size to 0.2 (0.2 is a level generally regarded as representing a clinically weak to negligible effect for psychological interventions).

The larger effect size found using the usually more conservative random effects compared to the fixed effects analysis is difficult to explain as a correction is made for small sample size bias. Wider confidence intervals are expected for the random effects analysis as they include estimates both of between and within study sampling variation.

### *Variables influencing effects (Figures 3 and 4)*

The marked heterogeneity of the data supported a preliminary

*© 1999 Cancer Research Campaign*

| Author & Year | | Treatment mode | *n** | R |
|---|---|---|---|---|
| Bloom | 1978 | Individ | 39 | – |
| Maguire | 1980 | Individ | 152 | + |
| West (T) | 1980 | Individ | 14 | + |
| Hayes (T) | 1981 | Group | 12 | – |
| Spiegel | 1981 | Group | 30 | + |
| Linn | 1982 | Individ | 92 | + |
| Price (T) | 1982 | Group | 44 | – |
| Christensen | 1983 | Couple | 20 | + |
| Youssef | 1983 | Group | 18 | – |
| Guerra. (T) | 1984 | Group | 34 | + |
| Worden | 1984 | Individ | 117 | – |
| Frankel (T) | 1985 | Group | 24 | + |
| Cain | 1986 | Individ | 72 | + |
| Houts | 1986 | Individ | 32 | – |
| Hurst (T) | 1986 | Group | 23 | + |
| Telch | 1986 | Group | 27 | + |
| Fawzy | 1990 | Group | 66 | + |
| Bindemann | 1991 | Relax | 71 | + |
| Decker | 1992 | Relax | 63 | + |
| Greer | 1992 | Individ | 151 | + |
| **Total** | | | **1101** | |

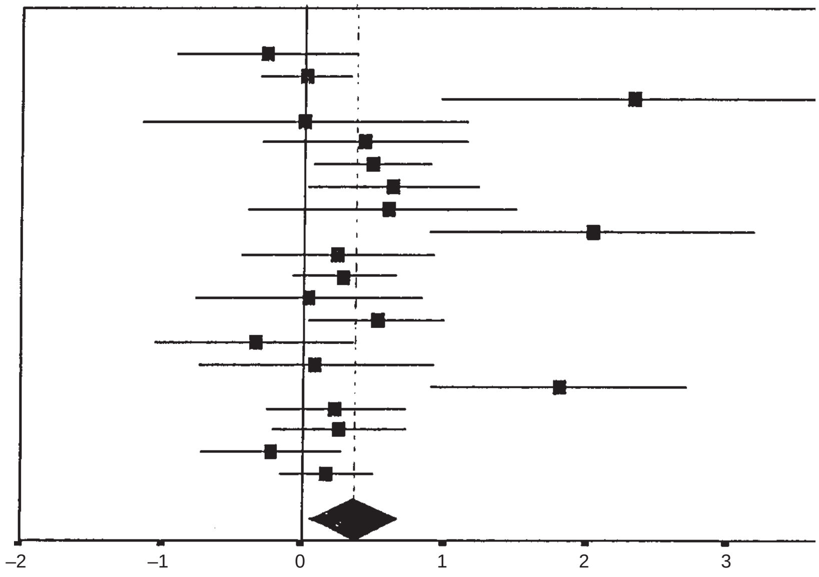

(T) = Unpublished thesis
R = Randomized assignment
* For trials with more than one intervention group (Cain, Davis and Telch) sample size is the number in the included experimental group plus the number in the control group

**Figure 5** Effect sizes and 95% confidence intervals for individual studies: depression

| | *n** | ES*** |
|---|---|---|
| Published | 14 | 0.34 |
| Unpublished | 5** | 0.27 |
| More reliable design | 8 | 0.21 |
| Less reliable design | 12 | 0.50 |
| Two extreme positive outliers removed | 17 | 0.19 |
| Fixed effects analysis | 20 | 0.25 |
| Random effects analysis | 20 | 0.36 |

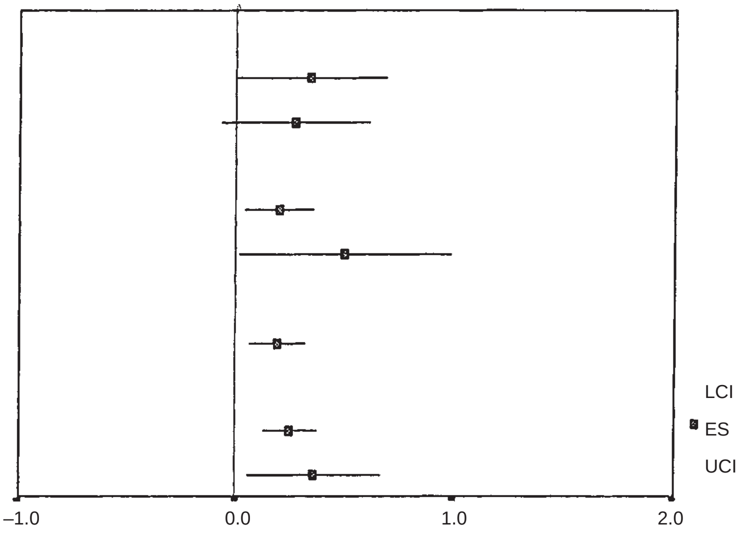

* *n* = Number of trials
** Excluding one extreme outlier (West)
*** ES = Effect size (All random effects analysis with exception of fixed effects statistic) Vertical line is zero

**Figure 6** Sensitivity analysis: depression exploration of the possible moderating effect of clinically relevant variables. This was done using hypotheses formed before beginning the data analysis. Contrary to prediction interventions delivered in an individual format had an effect size similar to relaxation alone and only approximately 50% that of interventions in a group format ($P = 0.0076$). Three trials of groups psycho-educational courses (Johnson, 1982; Telch and Telch, 1986; Fawzy et al, 1996) had a considerably larger mean effect than other group interventions ($P = 0.0005$). This subgroup almost entirely accounts for the difference in effect size between group and individual interventions (Figure 3); it also contains both of the positive outliers. Further analysis of the influence of type of therapy on effect size could have been of considerable clinical and theoretical interest but was precluded by the great diversity of types of intervention.

The findings for other variables which might influence effect

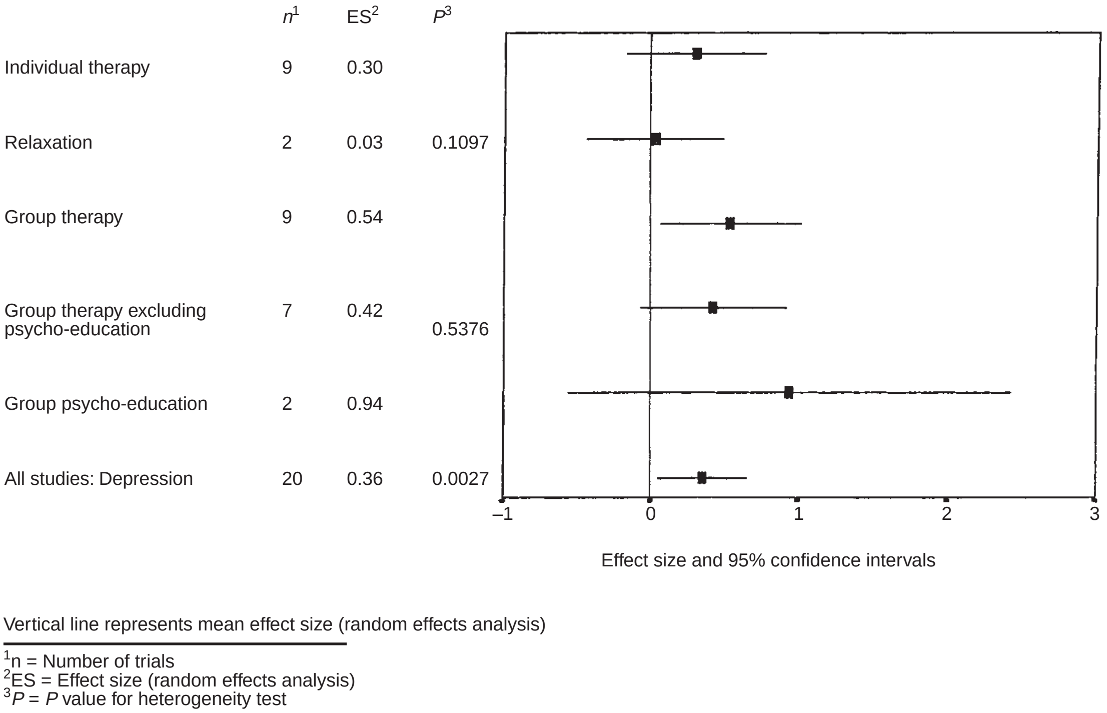

**Figure 7** Treatment type: depression

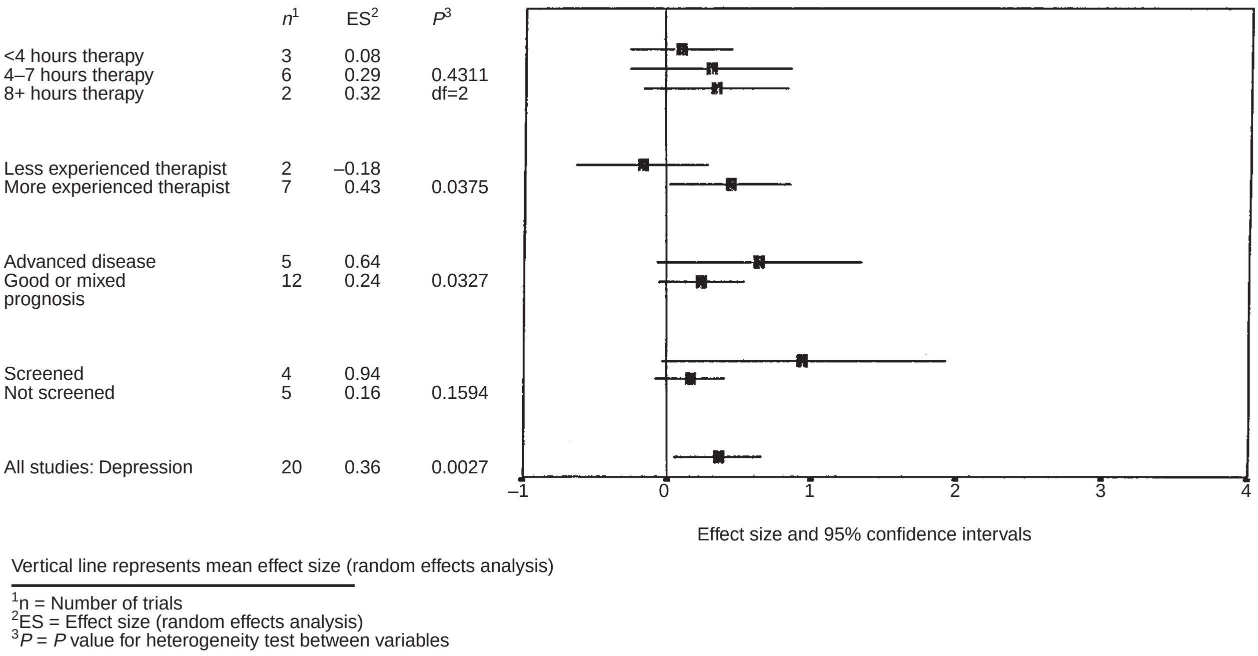

**Figure 8** Dose, therapist and subject variables: depression

(Figure 4) must be viewed with considerable caution as they are based only on those studies, on occasion a small minority, which actually specified them and therefore cannot be taken as representative of the sample. The data suggest a dose–response effect ($P = 0.0017$) which is unlikely to be due to a maturation confound as interventions given over a 6-week or shorter period actually have a marginally greater effect size (0.6) than those taking longer than 6 weeks (0.52). The use of more experienced therapists is associated with a larger effect but this falls just below the 5% level ($P = 0.054$). Effects were preserved at follow-up in the small minority ($n = 4$) of trials which examined this important outcome at a variety of time points beyond the end of the intervention (Cain

 *© 1999 Cancer Research Campaign*

et al, 1986; Davis, 1986; Fawzy et al, 1990; Greer et al, 1992).

Unfortunately there was insufficient data available to explore the potentially important influence of the timing of the commencement of interventions relative to diagnosis, relapse and treatment.

### Depression meta-analysis

Twenty trials were included (see Table 1). A total of 30 trials were identified which measured depression and met initial entry criteria, but ten were excluded as effect sizes could not be estimated (Table 2). Figure 5 shows the spread and confidence intervals for the 20 trials. The total sample size is 1101, the combined effect size is 0.36 and the dataset is strongly heterogeneous. Twelve depression effect sizes were measured using the Profile of Mood States (POMS) depression subscale (McNair et al, 1971), five using the Beck Depression Inventory (Beck and Steer, 1987) and three used other measures.

#### *Sensitivity analysis (Table 2 and Figure 6)*

The ten excluded studies are summarized in Table 2. Inclusion of four (Farash, 1977; Gordon et al, 1980; Bridge et al, 1988; Burton and Parker, 1988) would probably have reduced the mean effect size, while inclusion of one (Watson et al, 1988) would have increased it.

Removal of three positive outliers (West, 1980; Youssef, 1984; Telch and Telch, 1986) with a total sample size of 59 reduces the overall effect by a half to 0.19 (Figure 6).

The use of randomization did not appear to influence effect: the 14 randomized trials have a very similar combined effect size to the six non-randomized trials (0.38 vs 0.31). Superior study quality appears to be associated with weaker effects (Figure 2): eight studies meeting the criteria for more reliable design have a smaller mean effect size than 12 less reliable studies (0.21 vs 0.50). However, this difference is entirely attributable to the three positive outliers falling into the unreliable design group. Their elimination reduces the combined effect size for this group to 0.18, equivalent to the 0.21 value for the more reliable studies.

Publication bias does not appear to be a feature of the published subsample as these trials have very similar mean effect sizes to the unpublished trials even with one unpublished small sample size extreme positive outlier (West, 1980) removed. A funnel plot does not show a skewed distribution. Sixteen unpublished studies with an effect size of zero hidden in researchers' filing cabinets are required to reduce the mean effect size to 0.2.

#### *Variables influencing effects (Figures 7 and 8)*

The marked heterogeneity of the data supported a preliminary exploration of the possible moderating effect of clinically relevant variables. As for the anxiety data-set individual interventions have a smaller effect size than group interventions but for depression this difference is not statistically significant. The two trials of relaxation have an aggregate effect size of zero, one had a positive effect (Bindemann et al, 1991), one a negative effect (Decker et al, 1992).

Again the findings for other variables which might influence effect must be viewed with considerable caution as they represent data only from those studies which actually specified them. As for anxiety a larger effect size is associated with higher therapist level of training and experience in oncology ($P = 0.0375$ with one very small sample size extreme outlier removed (West, 1980)). Effect size is greater for those with advanced disease ($P = 0.0327$). Only three trials included follow-up measures (Halttunen et al, 1992), mean effect at post-test (0.27) was at least sustained at follow-up (0.49).

### DISCUSSION

As expected the included trials show considerable variation in subjects, settings, intervention modality, theoretical base, therapist expertise, amount of therapy given and experimental methods. This clinical marked heterogeneity suggests that the emphasis in these meta-analyses should be on the main effects, in particular their clinical significance and robustness.

The anxiety main effect of 0.42 can be taken to be fairly robust. Publication bias in the published sub-sample appears to have been corrected by inclusion of unpublished theses which had smaller effects and sample sizes. Twenty unpublished trials of zero effect size would be required to produce a 50% reduction in effect size. Inclusion of the seven studies which were excluded because of missing data would most probably have had very little impact on the combined effect size. The main threat to robustness appears to be the large influence of two positive outliers with a combined sample size of 79. Removing them renders the overall effect much smaller and of marginal clinical significance. However, trials with more reliable design have a moderate to strong effect size of 0.63. Only one of the extreme positive outliers falls into this group and its removal reduces the effect size down to 0.36; this is comparable with, but slightly less than the overall effect size of 0.42 for all 19 anxiety studies. This value of 0.36, based on the ten of the 19 trials of most reliable design, can be taken to be the most robust summary statistic for the anxiety trials.

Normative data for the Spielberger State Anxiety Inventory (STAI), which was used in five of the 19 trials, give some indication of the clinical significance of these anxiety effect sizes. A 0.36 standard deviation shift for the STAI is approximately equivalent to the difference between the anxiety levels of normal subjects and that of general medical and surgical in-patients (Spielberger et al, 1983).

The depression main effect of 0.36 is not as robust as that for anxiety: there is no evidence of publication bias but including only those trials with more reliable design decreases it by nearly 50% to 0.21. Similarly, eliminating the three extreme positive outliers (which all fall into the less reliable design subgroup) reduces the mean effect to 0.19. Also inclusion of the trials excluded through missing data (Table 2) would also probably reduce the mean effect. The Beck Depression Inventory (BDI) was used in five of the 20 depression trials; for the BDI a 0.36 standard deviation shift approximates to 50% (and 0.21 standard deviation to 30%) of the difference between the means of moderate and mild depression (Beck et al, 1961), but a 0.21, or 0.19 shift is little more than half of this and of very doubtful clinical significance. In summary, the main effect for anxiety can be taken to be of moderate clinical significance, that for depression as weak to negligible.

The clinically significant effect for anxiety but not depression may in part be attributable to the well-established finding that the prevalence of anxiety in oncology populations is greater than that for depression (Parle et al, 1996). Prevalence is relevant in this case as the great majority of both the anxiety and depression trials in this sample were preventative in nature, i.e. the subjects were not selected on psychological criteria. This preventative orientation is a very important feature of the trials included in these two

*© 1999 Cancer Research Campaign*

meta-analyses; the majority of trials recruited subjects on the basis of a diagnosis of cancer, or being thought suitable for inclusion by an oncologist, or being self-referred. Consequently, a large proportion of the individuals receiving an intervention may have neither needed nor benefited from it. This would substantially reduce effect size and indeed the mean effect even for anxiety is only about 50% of the mean effect of 0.69 found in a meta-analysis of trials of psychotherapy for depression (Robinson et al, 1990) (not cancer patients). However, the anxiety effect is not a great deal smaller than the overall mean of 0.5 found by Lipsey and Wilson (1993) in their meta-analysis of meta-analyses of psychological interventions in many settings. Also four meta-analyses of antidepressants for depression have reported effect sizes between 0.19 and 0.79 (Smith et al, 1980; Shapiro and Shapiro, 1982; Quality Assurance Project, 1983; Steinbrueck et al, 1983; Greenberg et al, 1992).

Unfortunately, only four trials specifically recruited subjects identified as either suffering from, or at high risk of, significant psychological distress (Linn et al, 1982; Worden and Weisman, 1984; Telch and Telch 1986; Greer et al, 1992). The effect sizes for screened subjects are large and similar for anxiety (0.94, $n = 3$) and depression (0.85, $n = 4$). For non-screened subjects the anxiety effect size is moderate to weak (0.33) but again the depression effect size is small and clinically insignificant (0.16) (Figure 4 and 8). These differences are not statisticaly significant at the 5% level, but the statistical power is low. More data is needed on the effectiveness of psychological interventions with cancer patients who are identified as either suffering from, or at risk of significant psychological distress.

The effect size for the anxiety meta-analysis is appreciably greater than that of 0.24 found by Meyer and Mark (1995) in their less focused fixed effects meta-analysis of 45 published trials of psychological interventions in oncology. Their sample included a wide range of psychological, educational or nursing interventions aimed at altering an extremely broad range of outcomes which included pain or treatment side-effects, psychological distress, identified problems and quality of life. The effect size of 0.24 was an average of any psychological outcome measures made in this disparate set of trials. We compared our samples with theirs: Meyer and Mark (1995) included only 11 of our 16 published trials (and by definition none of our nine unpublished trials), and only one (80) of our ten trials (two unpublished) excluded because of missing data. They did not appear to have identified any trials unknown to us. Meyer and Mark's findings are therefore based on a sample which is 75% different from ours, less focused in terms of trial aims and outcomes, and which appears to be less representative of the literature. Most of the difference in findings could be attributed to these factors.

Examination of extreme outliers is important as they may represent an important subset with particular features in common which account for their distance from the mean. For anxiety there are two obvious positive outliers which are both trials of group psycho-educational courses (Johnson, 1982; Telch and Telch, 1986). This type of intervention has a large mean effect size of 1.59 ($n = 3$ trials, total sample size 145) compared with 0.27 found for other group interventions. There was no obvious pattern to the three depression-positive outliers which consisted of trials of group therapy (Capone et al, 1980), of a group psycho-educational course (69) and individual cognitive therapy (Linn et al, 1982). There were no obvious negative outliers, but six anxiety and three depression trials did have negative effect sizes. This statistical variation is expected but the majority of these trials also had features which made a negative finding more likely.[1]

1 Three trials were common to the anxiety and depression samples and the negative effect can be attributed to the intervention groups being considerably more distressed at baseline (Bloom et al, 1978; Davis, 1986; Decker et al, 1992). In the other three anxiety trials one used a post-test only design (Cumbia, 1985), one was an evaluation of the effect of a brief telephone intervention given to subjects already receiving a psychosocial care programme (Houts et al, 1986), and one had no features that might obviously account for the intervention group faring worse than the controls (Hurst, 1986).

### Interaction effects

Exploration of the effect of therapy type produced unexpected findings (Figures 3 and 7). The large effects found for the few trials of group psycho-educational courses for both anxiety and depression are impressive but require replication. The equivalent effect of group and individual therapy (after subtraction of group psycho-educational courses) was contrary to prediction and contrasts with Shapiro and Shapiro's meta-analysis of psychotherapy which found individual therapy to be more effective than group (Shapiro and Shapiro, 1982). This unusual finding could be a reflection of cancer patients having a shared predicament. The data on relaxation are sparse but as predicted there was an effect on anxiety but not depression. The influence of other hypothesized moderator variables is only suggestive and is presented (Figures 4 and 8) mainly as a potential basis for hypothesis generation for future trials or explanatory meta-analyses. The numbers of studies in some subgroups are extremely small but the results are strengthened by their being very largely in the expected direction.

The findings have considerable clinical and service implications:

i. These data can help inform the problem of where best to direct limited clinical resources. The results indicate that preventative routine psychological intervention for all cancer patients will at best have a clinically moderate effect on anxiety, but a negligible one on depression. Resources are scarce and therefore it is likely that clinically powerful and therefore cost-effective outcomes for anxiety and depression are only likely to result in interventions targeted at those suffering from or at risk of significant psychological distress. However, only four out of the 25 trials in the two meta-analyses intervened specifically on such patients. More trials of this kind are needed to establish whether they reliably result in clinically strong effects.

ii. Group therapy appears equally effective to individual, or perhaps considerably more so in the case of group psycho-educational courses. Group therapy is clearly cheaper to provide but the group therapy data are exclusively North American and therefore replication in the very different European cultural and health service context is required.

iii. There is evidence to suggest that relatively short but intensive interventions delivered by experienced and more highly trained therapists are more effective than more protracted interventions offered by less psychologically trained staff. It is possible that interventions of longer duration carry the risk of over-sensitizing subjects to their status as cancer patients, leading them to doubt their ability to cope without professional support (Maguire et al, 1980). However, more data are required on longer term outcome.

*© 1999 Cancer Research Campaign*

### SUMMARY

### Main effects

A main effect of 0.36 for anxiety can be taken to be of moderate clinical significance, this estimate is robust for a number of factors. The main effect for depression is not robust, taking this into account renders it clinically weak to negligible at 0.19–0.21.

### Clinical and service implications

Clinically strong and cost-effective outcomes are likely to result from interventions targeted at those suffering from or at risk of significant psychological distress. However, more data are needed to confirm this suggestion. Group interventions, particularly psycho-educational courses, are at least as effective as individual. If this finding can be replicated in Europe then group interventions should prove considerably more cost effective than individual (Fawzy et al, 1996).

### Research implications

Routine use of randomization and samples large enough to provide adequate statistical power will improve the reliability of future trial data. We now have adequate estimates of the overall magnitude of effect of psychological interventions on anxiety and depression in oncology. Consequently, future research would be best directed to specific questions, the analyses suggest current priorities as being establishing:

a. the effectiveness of interventions targeted at those at risk of, or suffering significant distress.
b. the viability and effectiveness of group therapy in European oncology settings.
c. whether the large effects associated with group psycho-educational courses can be replicated.
d. whether positive effects are maintained at long-term follow-up.

### ACKNOWLEDGEMENTS

We would particularly like to thank Lesley Stewart (Meta-analysis group co-ordinator) and Mahesh Parmar (Senior Statistician) of the Meta-analysis Group of the MRC Cancer Trials Office in Cambridge and the Cancer Research Campaign who funded this project. We would also like to thank Paschal Sheeran, Department of Psychology, University of Sheffield, Beverley Jones, CRC Psychological Medicine Group, Christie Hospital, Manchester, and many patient and helpful librarians in: Christie Hospital Medical Library, Blackberry Hill Hospital Library, University of Bristol Medical School and Arts and Social Sciences Libraries.

### REFERENCES

Andersen BL (1992) Psychological interventions for cancer patients to enhance quality of life. *J Consult Clin Psychol* **60**: 552–568

Beck AT and Steer RA (1987) *Beck Depression Inventory Manual*. The Psychological Corporation

Beck AT, Ward CH, Mendelson M, Mock J and Erbaugh J (1961) An inventory for measuring depression. *Arch Gen Psychiatry* **4**: 53–63

Bindemann S, Soukop M and Kaye SB (1991) Randomised controlled study of relaxation training. *Eur J Cancer* **27**: 170–174

Bloom JR, Ross RD and Burnell G (1978) The effect of social support on patient adjustment after breast surgery. *Pat Counsell Health Educ* **1**: 50–59

Brennan JH and Sheard TAB (1994) Psychosocial support and therapy in cancer care. *Eur J Palliat Care* **1**: 136–139

Bridge LR, Benson P, Pietroni PC and Priest R (1988) Relaxation and imagery in the treatment of breast cancer *Br Med J* **297**: 1169–1172

Burton MV and Parker RW (1988) A randomized controlled trial of preoperative psychological preparation for mastectomy. A preliminary report. In: *Psychosocial Oncology*, Watson M, Greer S and Thomas C (eds). Pergamon: Oxford

Cain EN, Kohorn EI, Quinlan DM, Latimer K and Schwartz PE (1986) Psychosocial benefits of a cancer support group. *Cancer* **57**: 183–189

Capone MA, Good RS, Westie KS and Jacobson AF (1980) Psychosocial rehabilitation of gynaecologic oncology patients. *Arch Phys Med Rehabil* **61**: 128–132

Carpenter JG (1984) A study of group psychosocial intervention with cancer patients and their families. PhD Thesis, Virginia Commonwealth University, USA

Christensen DN (1983) Postmastectomy couple counselling: An outcome study of a structured treatment protocol. *J Sex Marital Ther* **9**: 266–275

Clacey R, Thomas C and Pearson H (1988) Does counselling by nurses for mastecomy patients work? In: *Psychosocial Oncology*, Watson M, Greer S and Thomas C (eds). Pergamon: Oxford

Cook TD and Campbell DT (1979) *Quasi Experimentation: Design and Analysis Issues for Field Settings*. Houghton Miflin: Boston

Cumbia GG (1985) Therapeutic intervention in the treatment of adult patients with metastatic cancer: a comparative study of two group counselling approaches. D Ed Thesis, College of William and Mary, Virginia, USA

Cunningham AJ (1988) From neglect to support to coping: the evolution of psychosocial intervention for cancer patients. In: *Stress and Breast Cancer*, Cooper CL (ed). Wiley: Chichester

Davis H (1986) Effects of biofeedback and cognitive therapy on stress in patients with breast cancer. *Psychol Rep* **59**: 967–974

Decker TW, Cline-Elsen J and Gallagher M (1992) Relaxation therapy as an adjunct in radiation oncology. *J Clin Psychol* **48**: 388–393

Derogatis LR, Morrow GR, Fetting D, Penman S, Piasetsky S, Schmale AM, Henrichs M and Carnicke CLM (1983) The prevalence of psychiatric disorders among cancer patients. *JAMA* **249**: 751–757

Devlen J, Maguire P, Phillips P, Crowther D and Chambers H (1987) Psychological problems associated with diagnosis and treatment of lymphomas. *Br Med J* **295**: 953–957

Fairbanks CA (1981) The effects of psychological intervention on the psychological and survival status of advanced cancer patients. PhD Thesis, Florida School of Professional Psychology, USA

Fallowfield L (1995) Psychosocial interventions in cancer should be part of every patient's management plan. *Br Med J* **311**: 1316–1317

Fallowfield LJ (1988) Counselling for patients with cancer. *Br Med J* **297**: 727–728

Fallowfield LJ and Roberts R (1992) Cancer counselling in the United Kingdom. *Psychol Health* **6**: 107–117

Farash JL (1977) Effect of counselling on resolution of loss and body image disturbance following a mastectomy. PhD Thesis, California School of Professional Psychology, Los Angeles, USA

Fawzy FI, Cousins N, Fawzy NW, Kemeny ME, Elashoff R and Morton D (1990) A structured psychiatric intervention for cancer patients: 1. Changes over time in methods of coping and affective disorders. *Arch Gen Psychiatry* **47**: 720–725

Fawzy FI, Fawzy NW and Wheeler JG (1996) A post-hoc comparison of the efficiency of a psychoeducational intervention for melanoma patients delivered in group versus individual formats: an analysis of data from two studies. *Psycho-Oncology* **5**: 81–90

Frankel HB (1985) The effectiveness of a psychosocial intervention with cancer patients. PhD Thesis, Kent State University, USA

Golonka LM (1976) The use of group counselling with breast cancer patients receiving chemotherapy. D Ed Thesis, State University of New York, Albany USA

Gordon WA, Freidenbergs I, Diller L, Hibbard M, Wolf C, Levine L and Lucido D (1980) Efficacy of psychosocial intervention with cancer patients. *J Consult Clin Psychol* **48**: 743–759

Greenberg RP, Bornstein RF, Greenberg MD and Fisher S (1992) A meta-analysis of antidepressant outcome under "blinder" conditions. *J Consult Clin Psychol* **60**: 664–669

Greer S, Moorey S, Baruch JDR, Watson M, Robertson BM, Mason A, Rowden L, Law MG and Bliss JM (1992) Adjuvant psychological therapy for patients with cancer: A prospective randomised trial. *Br Med J* **304**: 675–680

Guerrant D (1984) Attitude adjustment of cancer patients after treatment based on a holistic model. PhD Thesis, Florida Institute of Technology, USA

© 1999 Cancer Research Campaign 

Halttunen A, Hietanen P, Jallinoja P and Lonnqvist J (1992) Getting free of cancer: an eight-year perspective of the relapse free patients. *Acta Oncol* **31**: 307–310

Harman MJ (1991) The use of group psychotherapy with cancer patients: a review of recent literature. *J Spec Group Work* **16**: 56–61

Hayes F (1981) The effects of the Simonton's methods including relaxation and imagery training in alleviating the psychological state of depression characteristic of cancer patients. PhD Thesis, Florida School of Professional Psychology, USA

Hedges LV and Olkin I (1985) *Statistical Methods for Meta-analysis*. Academic Press: Orlando

Heinrich RL and Coscarelli Schag C (1985) Stress and activity management: group treatment for cancer patients and spouses. *J Consult Clin Psychol* **53**: 439–446

Hopwood P and Maguire P (1992) Priorities in the psychological care of cancer patients. *Int Rev Psychiatry* **4**: 35–44

Houts PS, Whitney CW, Mortel R and Bartholomew MJ (1986) Former cancer patients as counsellors of newly diagnosed cancer patients. *J Natl Cancer Inst* **76**: 793–796

Hurst DF (1986) An adaptation of a psychoeducational program for individuals with cancer to a small group. PhD Thesis, Indiana State University, USA

Johnson J (1982) The effects of a patient education course on persons with a chronic illness. *Cancer Nurs* **5**: 117–123

Linn MW, Linn BS and Harris R (1982) Effects of counselling for late stage cancer patients. *Cancer* **49**: 1048–1055

Lipsey MW and Wilson DB (1993) The efficacy of psychological, educational and behavioural treatment: confirmation from meta-analysis. *Am Psychol* **48**: 1181–1209

Maguire P, Tait A, Brooke M, Thomas C and Sellwood R (1980) Effect of counselling on the psychiatric morbidity associated with mastectomy. *Br Med J* **281**: 1454–1456

Massie M and Holland J (1990) Overview of normal reactions and prevalence of psychiatric disorders. In: Holland J and Rowland J. *Handbook of Psycho-oncology*, pp. 273–282. OUP: New York

McNair DM, Lorr M and Dropplemann LF (1971) *Manual, Profile of Mood States*. Educational and Industrial Testing Service: San Diego

Meyer TJ and Mark MM (1995) *Health Psychol* **14**: 101–108

Parle M, Jones B and Maguire P (1996) Maladaptive coping and affective disorders among cancer patients. *Psychol Med* **26**: 735–744

Price RG (1982) An evaluation of a psychotherapeutic program for cancer patients in terms of depression. PhD Thesis, United States International University, USA

Quality Assurance Project (1983) A treatment outline for depressive disorders. *Aus NZ J Psychiatry* **17**: 129–146

Raudenbush SW (1994) Random effects models. In: *The Handbook of Research Synthesis*, Cooper H and Hedges LV (eds). Russel Sage Foundation: New York

Robinson LA, Berman JS and Neimeyer RA (1990) Psychotherapy for the treatment of depression: A comprehensive review of controlled outcome research. *Psychol Bull* **108**: 30–49

Rosenthal R (1979) The file drawer problem and tolerance for nul results. *Psychol Bull* **86**: 638–641

Shapiro DA and Shapiro D (1982) Meta-analysis of comparative therapy outcome studies: A replication and refinement. *Psychol Bull* **92**: 481–604

Smith ML, Glass GV and Miller TI (1980) *The Benefits of Psychological Therapy*. Johns Hopkins University Press: Baltimore

Spiegel D, Bloom JR and Yalom I (1981) Group support for patients with metastatic breast cancer: A randomised prospective outcome study. *Arch Gen Psychiatry* **38**: 527–533

Spielberger CD, Gorsuch RL, Luschene R, Vagg PR and Jacobs GA (1983) *Manual for the State-trait Anxiety Inventory*. Consulting Psychologists Press: Palo Alto

Steinbrueck SM, Maxwell SE and Howard GS (1983) A meta-analysis of psychotherapy and drug therapy in the treatment of unipolar depression with adults. *J Consult Clin Psychol* **51**: 856–863

Telch CF and Telch MJ (1986) Group coping skills instruction and supportive group therapy for cancer patients: a comparison of strategies. *J Consult Clin Psychol* **54**: 802–808

Thompson SG (1993) Controversies in meta-analysis: the case of the trials of serum cholesterol reduction. *Stat Methods Med Res* **2**: 173–192

Trijsburg RW, van Knippenberg FCE and Rijpma SE (1992) Effect of psychological treatment on cancer patients: a critical review. *Psychosom Med* **54**: 489–517

Vachon MLS (1988) Counselling and psychotherapy in paliative/hospice care: a review. *Palliat Med* **2**: 36–50

Watson M (1983) Psychosocial intervention with cancer patients: a review. *Psychol Med* **13**: 839–846

Watson M, Denton S, Baum M and Greer S (1988) Counselling breast cancer patients: a specialist nurse service. *Counsell Psychol Quart* **1**: 25–33

West BL (1980) Cognitive behavioural analysis system of psychotherapy (C-BASP) as applied to depression in a cancer population. PhD Thesis, Virginia Commonwealth University, USA

Worden JW and Weisman AD (1984) Preventive psychosocial intervention with newly diagnosed cancer patients. *Gen Hosp Psychiatry* **6**: 243–249

Youssef FA (1984) Crisis intervention: a group-therapy approach for hospitalised breast cancer patients. *J Adv Nurs* **9**: 307–313

*© 1999 Cancer Research Campaign*
